# Modulating Role of Breastfeeding Toward Long COVID Occurrence in Children: A Preliminary Study

**DOI:** 10.3389/fped.2022.884962

**Published:** 2022-03-30

**Authors:** Giulia Vizzari, Daniela Morniroli, Valentina Tiraferri, Silvana Castaldi, Maria Francesca Patria, Paola Marchisio, Carlo Agostoni, Fabio Mosca, Danilo Buonsenso, Gregorio Paolo Milani, Maria Lorella Giannì, The CHICO Study Group, Francesco Folino

**Affiliations:** ^1^Fondazione IRCCS Ca' Granda Ospedale Maggiore Policlinico, Neonatal Intensive Care Unit (NICU), Milan, Italy; ^2^Department of Clinical Sciences and Community Health, University of Milan, Milan, Italy; ^3^Fondazione IRCCS Ca' Granda Ospedale Maggiore Policlinico, Milan, Italy; ^4^Department of Biomedical Sciences for Health, Postgraduate School of Public Health, University of Milan, Milan, Italy; ^5^Fondazione IRCCS Ca' Granda Ospedale Maggiore Policlinico, Pediatric Unit, Milan, Italy; ^6^Department of Pathophysiology and Transplantation, University of Milan, Milan, Italy; ^7^Department of Woman and Child Health and Public Health, Fondazione Policlinico Universitario “A. Gemelli”, Rome, Italy; ^8^Center for Global Health Research and Studies, Dipartimento di Scienze Biotecnologiche di Base, Cliniche Intensivologiche e Perioperatorie, Università Cattolica del Sacro Cuore, Rome, Italy

**Keywords:** human milk, childhood, immune system, COVID-19, breastfeeding

## Abstract

The SARS-CoV-2 (Severe acute respiratory syndrome Coronavirus-2) pandemic has forced the global health system to face new challenges both in the acute management of COVID-19 (Coronavirus Disease 2019) patients and in its consequences. In particular, the long-term effects of this new virus, especially in children, are still poorly understood. Scientific research is currently trying to understand the mechanisms underlying the so called “long COVID syndrome”. Since the beginning of the pandemic, breastmilk has been studied for its antiviral and immunomodulatory properties. Based on these assumptions, we conducted a preliminary study in order to investigate the prevalence of long COVID in a cohort of Italian children with previously detected SARS-CoV-2 infection and evaluate if breastfeeding might play a role in modulating long COVID occurrence.

## Introduction

Over the past 2 years, the COVID-19 pandemic has posed a significant risk to the health of all age groups. However, the pediatric population is at a lower risk of hospitalization and death due to the SARS-CoV-2 (Severe acute respiratory syndrome Coronavirus-2) (1).

On the other hand, children affected by the SARS-CoV-2 infection might develop the multisystem inflammatory syndrome (MIS-C) and the long COVID syndrome as long-term consequences (2). Multisystem inflammatory syndrome in children (MIS-C) is a life-threatening, immune-mediated disease that occurs approximately from 2 to 6 weeks after being infected by SARS-CoV-2 and is characterized by dermatologic, mucocutaneous, gastrointestinal and cardiac manifestations (2).

The long-term outcome related to SARS-CoV-2 infection called “long COVID” or “post COVID syndrome” has so far been described mainly for the adult population, given the higher incidence of infection in older age groups during the first waves of the pandemic (3, 4). However, due to the emergence of infectious variants and the relatively low COVID-19 vaccination coverage among children, the incidence of COVID-19 in children is increasing considerably, and long COVID syndrome is more and more observed in the pediatric population (5, 6). This long term syndrome occurs after a period of at least 12 weeks from SARS-CoV-2 infection and seems to be characterized by a large number of non-specific manifestations, such as fatigue, sleep and concentration difficulties, headache, loss of appetite, exercise intolerance, muscle or joint pain (5, 7). However, little is known about risk factors of long COVID syndrome in children (8). As well as known, breast milk has been highlighted as a possible protective factor from SARS-CoV-2 infection for its well-known antimicrobial and antiviral factors (9). Its numerous bioactive compounds with immunomodulatory and antiviral activity contribute to the development of the infant's immune system conferring protection against infections both in the short and long term (10). These bioactive factors include a large spectrum of bioactive compounds, such as antibodies, cytokines, polyunsaturated fatty acids, lactoferrin, mucins, human milk oligosaccharides (HMOs), microbiota, and microRNAs (11). Previous observations suggested that breastmilk might be protective against COVID-19 in childhood, but it is still unknown if it might be associated with the risk of long COVID development (12). We conducted a preliminary study in order to investigate the prevalence of long COVID in a cohort of Italian children with a history of SARS-CoV-2 infection and to evaluate if breastfeeding could play a role in modulating long COVID occurrence within this sample.

## Methods

We enrolled a population of children (≤18 years old) with diagnosis of SARS-CoV-2 infection confirmed by a molecular test, admitted to the Pediatric emergency department (ED) of the Fondazione IRCCS Ca' Granda Ospedale Maggiore Policlinico, Milan and the Fondazione Policlinico Universitario Gemelli, Rome, Italy between July 1, 2020, and June 31, 2021.

The study was conducted between November 01, 2021, and January 15, 2022. The study was approved by Institutional Ethics Committee. Caregivers of the recruited children were interviewed by a phone call after obtaining their informed consent to process the data. The each structured interview lasted about 15 min and was carried out by pediatricians. Before administration, the survey was pilot-tested among five caregivers and five pediatricians. The survey was composed of three sections and investigated the following items: (1) demographic data and educational level of the caregivers; (2) demographic data and gestational age of children, their health status, including the presence of chronic diseases, the clinical symptoms of COVID-19, if any, the need for hospitalization or intensive care, and the self or parent-reported development of symptoms suggestive for long COVID syndrome (defined as having persisting symptoms—never reported before COVID-19—such as dyspnea, mental confusion, fatigue, chest pain, problems associated to speech, anxiety and altered mood, muscular pain, fever, loss of taste and smell for at least 12 weeks); (3) mode of feeding during early infancy and duration of breastfeeding. The questionnaire is reported within the online supplementary material.

## Data Management and Statistical Analysis

All data were anonymously collected in a predefined, online database. Response rate was calculated as the number of subjects who accepted to participate, divided by the total number of subjects, including those who participated and those who denied the consent. Continuous data were reported as median and interquartile range (IQR) and discrete variables were expressed as absolute frequency and percentage. The association among children's basic characteristics (age, gender, gestational age, being affected by a chronic disease), symptomatic COVID-19, mode of feeding and long COVID occurrence was assessed using multivariate binary regression analysis. For analysis, age was categorized in two groups (≥10 vs. <10 years, respectively) since being ≥10 years old has been reported as a risk factor for long COVID occurrence.

## Results

Out of 132 caregivers contacted by phone, 121 accepted to fill the questionnaire and 11 declined to participate (response rate 92%). The main characteristics of the enrolled children and of their related caregivers are summarized in Table 1. Ten of the recruited children were moderately-late preterm neonates (8%). In our cohort, most children were breastfed, and more than half of them (55%) for more than 6 months. The median duration of any breastfeeding was 6 months (IQR 2–12) and of exclusive breastfeeding was 4 months (IQR 1–6). As far COVID-19 is concerned, three out of four children presented with symptomatic SARS-CoV-2 infection and even if 22% of these ones required hospital admission, no one needed intensive care. Suggestive symptoms of long-COVID syndrome were reported in 37% of all enrolled children. At the multivariate binary regression analysis, being older than 10 years old was independently associated with higher risk of long COVID occurrence whereas no association was found between long COVID and having been breastfed. Although not being statistically significant, having had symptomatic COVID-19 infection showed a trend toward being independently associated with a higher risk of long COVID occurrence (Table 2).

**Table 1:** Baseline characteristics of caregivers and children (data are given as median and interquartile range or absolute frequency and percentage).

**Caregiver**
Age—years, median (IQR)	42 (38–47)
Gender—female, *N* (%)	97 (80)
**Educational level—*N* (%)**
• Secondary school	19 (16)
• High school	36 (30)
• University	43 (35)
• Post university	23 (19)
**Child**
Age years, median (IQR)	9.0 (2.0–12.0)
Group of children ≥ 10 years old, N (%)	53 (44)
Gender—female, *N* (%)	48 (40)
Gestational age—weeks, median (IQR)	39 (38–40)
Underlying chronic disease—*N* (%)	18 (15)
**COVID-19 history**
Months from COVID-19 infection—median (IQR)	11 (6–13)
Symptomatic COVID-19—*N* (%)	89 (74)
Hospitalization due to COVID-19—*N* (%)	27 (22)
Need of intensive care due to COVID-19—*N* (%)	0 (0.0)
Long COVID—yes, *N* (%)	45 (37)
**Feeding's mode**
Breastfeeding—yes, *N* (%)	101 (84)

**Table 2:** Multivariate binary logistic regression predicting the likelihood of having long COVID (dependent variable).

**Variables**	**OR**	**Lower 95%CI**	**Upper 95%CI**	***P***
Child's age (≥10 vs. <10 years)	3.57	1.61	7.91	0.002
Child's gender (male vs. female)	0.71	0.31	1.60	0.415
Gestational Age (<37 vs. ≥37 weeks)	1.02	0.20	5.08	0.980
Presence of child's chronic disease (yes vs. no)	0.73	0.22	2.33	0.598
Symptomatic COVID-19 (yes vs. no)	2.53	0.97	6.64	0.058
Breastfeeding (yes vs. no)	1.54	0.48	4.84	0.461

## Discussion

The current pandemic situation underlines the importance of breast milk as a potential protective factor against COVID-19 as both in short and long-term outcomes (13). Breastmilk represents a complex system that confers immediate protection to the newborn against infections and at the same time modulates the development of his immune system, representing a biological advantage in terms of short and long-term health outcomes of the offspring (10). In addition, Verd et al. in a recent short report support the idea that breastfeeding confers prolonged protection against various viral infections, including SARS-CoV-2 (12). To the best of our knowledge, this is the first study investigating the possible role of having been breastfed in the development of long COVID syndrome. According to our findings, approximately one third of children with a documented SARS-CoV-2 infection had symptoms of long COVID syndrome. Contrary to our findings, a recent Danish cohort study of 37,522 children by Borsch et al. estimated the incidence of long COVID symptoms in 0.8% of children with definite SARS-CoV-2 infection (14). However, the authors did not consider symptoms such as concentration difficulties, headache, muscle/joint pain and nausea as long COVID symptoms. Remarkably, as shown in a recent review by Zimmermann et al., the lack of firm definitions for the long COVID syndrome, as well as the lack of studies with adequate control groups, makes it difficult to estimate the true incidence of this condition in the pediatric population (5). In addition, more than 200 symptoms have been proposed as characteristic for long COVID syndrome but, in several studies, these symptoms were also present in the control population. Overall, the prevalence of long COVID observed in our cohort is in line with the results of a previous systematic review that show that symptoms of long COVID might develop in 4–66% of children with a history of SARS-CoV-2 infection (5).The multiple regression analysis pointed out a higher risk of long COVID syndrome in older children and a trend of risk in children with symptomatic COVID-19. These data are in accordance with the findings of a large observational study by the Sechenov Stop COVID Research Team, which included more than 500 pediatric subjects (15). Our study did not identify any association between breastfeeding and the risk of developing long COVID syndrome. This result could be at least partially explained by the relatively small sample size and the high rates of breastfeeding in our study's population, together with a median breastfeeding duration in line with the international recommendations (16). On the other hand, it is possible that breastfeeding might play a significant contribution in preventing COVID-19 infection, but a lower role in preventing its complications. A further speculation could be related to the nature of the SARS-CoV-2 agent, relatively new as far contamination rates and wide individually-related responsiveness (including autoimmune mechanisms) that human milk has not yet developed the slow evolutive adaptation able to support a specific defense. These findings suggest at least two implications: (1) breastfeeding should continue to be promoted even during the pandemic for its potential to reduce viral infections including the SARS-CoV-2 one (17); (2) future studies should further explore the relationship between breastmilk and SARS-CoV-2 infection and its role on modulating COVID-19 manifestations. In conclusion, this preliminary study focuses on a crucial issue that requires further research, with larger and heterogeneous samples in order to fully investigate the possible link between breastfeeding and protection against not only acute COVID-19 but also its long-term effects.

## Data Availability Statement

The raw data supporting the conclusions of this article will be made available by the authors, without undue reservation.

## Ethics Statement

The studies involving human participants were reviewed and approved by Policlinico Agostino Gemelli Roma informed consent to participate in this study was provided by the participants' legal guardian/next of kin.

## Chico Study Group

Francesco Folino, Maria Carola Pensabene, Marina Macchi, Cristina De Rose, Rosa Morello, Carolina Gentili, Margherita Zona, Alessia De Matteis.

## Author Contributions

DB, SC, CA, FM, MG, PM, and GM conceptualized and designed the study. GV and VT collected the data. MG and GM performed the statistical analyses. GV and DM writing original draft preparation. MG, GM, CA, FM, PM, and MP writing review and editing. All authors gave a significant contribution to data interpretation in their field of expertise, reviewed the manuscript, and approved it as submitted.

## Funding

This study was partially funded by a Grant of the Italian Ministry of Health Ricerca Corrente 2020.

## Conflict of Interest

The authors declare that the research was conducted in the absence of any commercial or financial relationships that could be construed as a potential conflict of interest.

## Publisher's Note

All claims expressed in this article are solely those of the authors and do not necessarily represent those of their affiliated organizations, or those of the publisher, the editors and the reviewers. Any product that may be evaluated in this article, or claim that may be made by its manufacturer, is not guaranteed or endorsed by the publisher.
